# Prevention of febrile neutropenia in diffuse large B-cell lymphoma treated with R-CHOP

**DOI:** 10.2478/abm-2025-0020

**Published:** 2025-09-02

**Authors:** Asian Biomedicine

**Affiliations:** Faculty of Medicine, Chulalongkorn University, Bangkok 10330, Thailand

Diffuse large B cell lymphoma (DLBCL) is the most common aggressive subtype of non-Hodgkin lymphoma (NHL), accounting for nearly 30% of all cases of NHL, and is phenotypically and genetically heterogeneous [1]. Patients usually present with a rapidly growing tumor mass in single or multiple, nodal, or extranodal sites such as nodes in the neck or abdomen [2]. Therefore, DLBCL should be investigated in patients with a nodal or extranodal mass. A summary of strategies to identify high-risk patients with DLBCL using clinical and biological prognostic factors has been proposed [3]. The diagnosis is based on characteristic morphology and immunophenotyping of an excisional or incisional lymph node biopsy.

DLBCL can be rapidly progressive but potentially curable with a combination of first-line drugs which include rituximab, cyclophosphamide, hydroxydaunorubicin or doxorubicin, oncovin or vincristine and prenisolone (R-CHOP) [4]. The effectiveness of different R-CHOP regimens has been tested in population-based studies [5]. Some patients with DLBCL may not be cured with the standard R-CHOP regimen. These patients may develop poor subsequent outcomes [6].

Febrile neutropenia (FN) is a serious complication of patients with DLBCL receiving R-CHOP [7]. In this issue, Nakaphan and Uaprasert [8] reported the effects of primary granulocyte colony-stimulating factor (G-CSF) prophylaxis for chemotherapy-induced FN in DLBCL patients receiving the R-CHOP-21. Their study highlights the high risk of FN among patients with DLBCL who receive R-CHOP-21. The authors advocated the use of primary G-CSF prophylaxis as a routine measure for the treatment of newly diagnosed DLBCL patients treated with R-CHOP-21 [8]. However, in patients with sarcopenia, the effectiveness of G-CSF in preventing FN in DLBCL treated with R-CHOP may be compromised [9]. The findings emphasize the need for collaborative efforts toward further advancing the treatment of DLBCL [10].
